# Pneumonia in the Pandemic: Not Always COVID-19

**DOI:** 10.7759/cureus.47802

**Published:** 2023-10-27

**Authors:** Nur Chandra Bunawan, Annisa D Harlivasari, Airin Aldiani

**Affiliations:** 1 Internal Medicine, Rumah Sakit Umum Daerah (RSUD) Kramat Jati, Jakarta, IDN; 2 Pulmonology, Rumah Sakit Umum Daerah (RSUD) Budhi Asih, Jakarta, IDN; 3 Pulmonology, Rumah Sakit Umum Daerah (RSUD) Kramat Jati, Jakarta, IDN

**Keywords:** tropical infections, covid-19, pandemic, : tuberculosis, covid-19 pneumonia

## Abstract

The coronavirus disease 2019 (COVID-19) pandemic has changed the way we manage patients, especially those with respiratory illnesses. Clinical manifestations, chest imaging, and reverse-transcription polymerase chain reaction (RT-PCR) play major roles in diagnosing respiratory infections during a pandemic. However, several infections can mimic COVID-19 regarding its clinical signs, symptoms, and imaging appearance. Diagnosing pneumonia other than COVID-19 is a big challenge in developing countries, given the limited resources available. We presented a case of a 25-year-old female with clinical symptoms and radiological characteristics typical of COVID-19 but a repeated negative RT-PCR test. Further workups found lung tuberculosis as her primary diagnosis. Our patient continued treatment with an antituberculosis agent and a short course of steroids, with a remission of symptoms.

## Introduction

The coronavirus disease 2019 (COVID-19) has changed the way people practice medicine worldwide. During the pandemic, every patient presenting with a symptom or sign of pneumonia will be suspected of COVID-19 [1]. Several diagnostic modalities can be used to pursue COVID-19 diagnosis, namely computed tomography scanning (CT scan), rapid antigen testing, rapid antibody testing, and nucleic acid testing [2].

Twice in a row, negative reverse polymerase chain reaction (RT-PCR) from the oropharyngeal and nasopharyngeal swabs is needed in order to discard the COVID-19 diagnosis, according to the national COVID task force in Indonesia [3]. However, problems emerged when the patient had classical symptoms and signs of COVID-19 but the RT-PCR test was negative. A repeated swab from a more sensitive sample (sputum, bronchial secret, or bronchoalveolar lavage) or paired sera of the COVID-19 antibody could be taken if the clinical suspicion for COVID-19 is still high, while other possible diagnoses should be considered [4].

Lung infections (influenza, cytomegalovirus, bacterial, fungal, *Pneumocystis jirovecii*, and tuberculosis), interstitial lung diseases, pulmonary edema, diffuse alveolar hemorrhage, pulmonary infarction, and pneumonia from vaping are some of the most common differential diagnoses of COVID-19, but extensive work-up is difficult to access in limited resource areas [5]. Here, we describe a case of a patient in a limited resource setting whose clinical and radiological symptoms were suggestive of COVID-19 pneumonia but turned out to be another disease entity.

## Case presentation

A 25-year-old woman was admitted due to shortness of breath for three days and a history of fever for one week, accompanied by a productive cough. Patients also had fatigue, nausea, and a loss of appetite. There was no previous medical history noted for the patient. Patients denied neither a history of traveling abroad nor close contact with COVID-19 patients. The patient has a history of incomplete COVID-19 vaccination with the Sinovac® vaccine.

**Figure 1:**
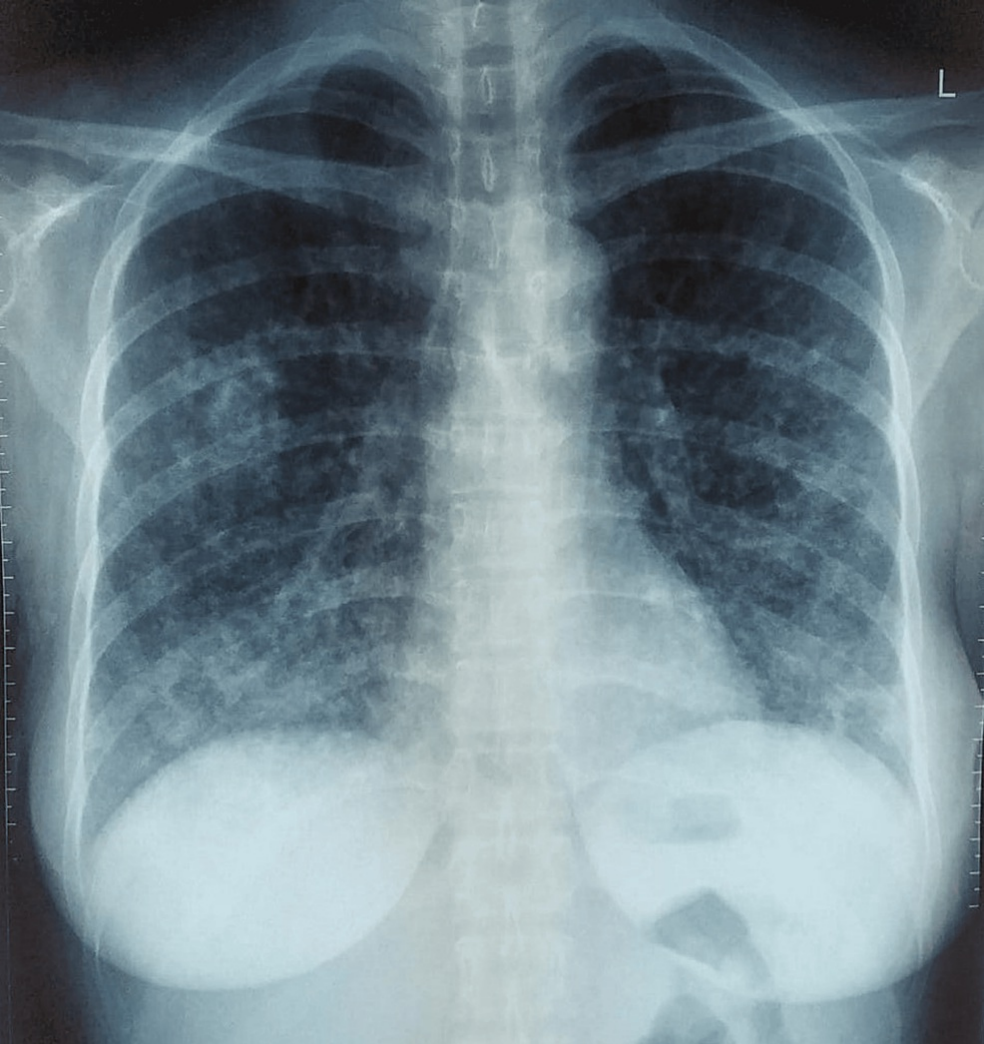
Chest X-ray in the primary hospital

From a physical examination, the patient appears in moderate distress with a temperature of 38.9 °C, a respiratory rate (RR) of 27 breaths/minute, and an oxygen saturation (SpO_2_) of 93% on room air. A physical examination showed crackles on both lung auscultations. There is an increased C-reactive protein level (CRP) of 81 mg/L. The chest X-ray showed bilateral opacities, especially in the peripheral zone, suggesting viral pneumonia (Figure 1). The antigen swab test for COVID-19 was negative. The patient was diagnosed with suspected COVID-19 pneumonia and underwent an oropharyngeal swab within the first two days of admission.

Therapy included oxygen at 3 lpm with a nasal cannula and the intravenous antibiotic ceftriaxone (2 g OD). After five days of hospitalization, the patient still had a fever with a temperature of 38.6 °C and shortness of breath. Both PCR results were negative for COVID-19 infection. There was a slight decrease in CRP level (53 mg/L) and d-dimer (5.53 ng/mL), and the anti-HIV test was non-reactive. The antibiotic was escalated to Meropenem 1 g TID (iv). Heparin prophylaxis was initiated on 10,000 units/24 jam (iv). We took the third nasopharyngeal and oropharyngeal swab test on day 6 of hospitalization, which also had a negative result. We could not do the bronchoscopy because it was not available in our hospital. Further history revealed the patient also had night sweats and unintentional weight loss for these two months. Rapid Molecular Test Xpert® MTB/RIF on a sputum sample was done with a positive result: Mycobacterium tuberculosis (MTB) was detected with no rifampicin resistance detected. Fixed Drug Combination (FDC) anti-tuberculosis medication, including Rifampicin, Isoniazid, Pyrazinamide, and Ethambutol, was given to the patient immediately. On day 8 of hospitalization, the patient's condition worsened. She became more dyspneic and was corrected with 7 lpm of a simple mask. The patient was referred to another hospital.

**Figure 2:**
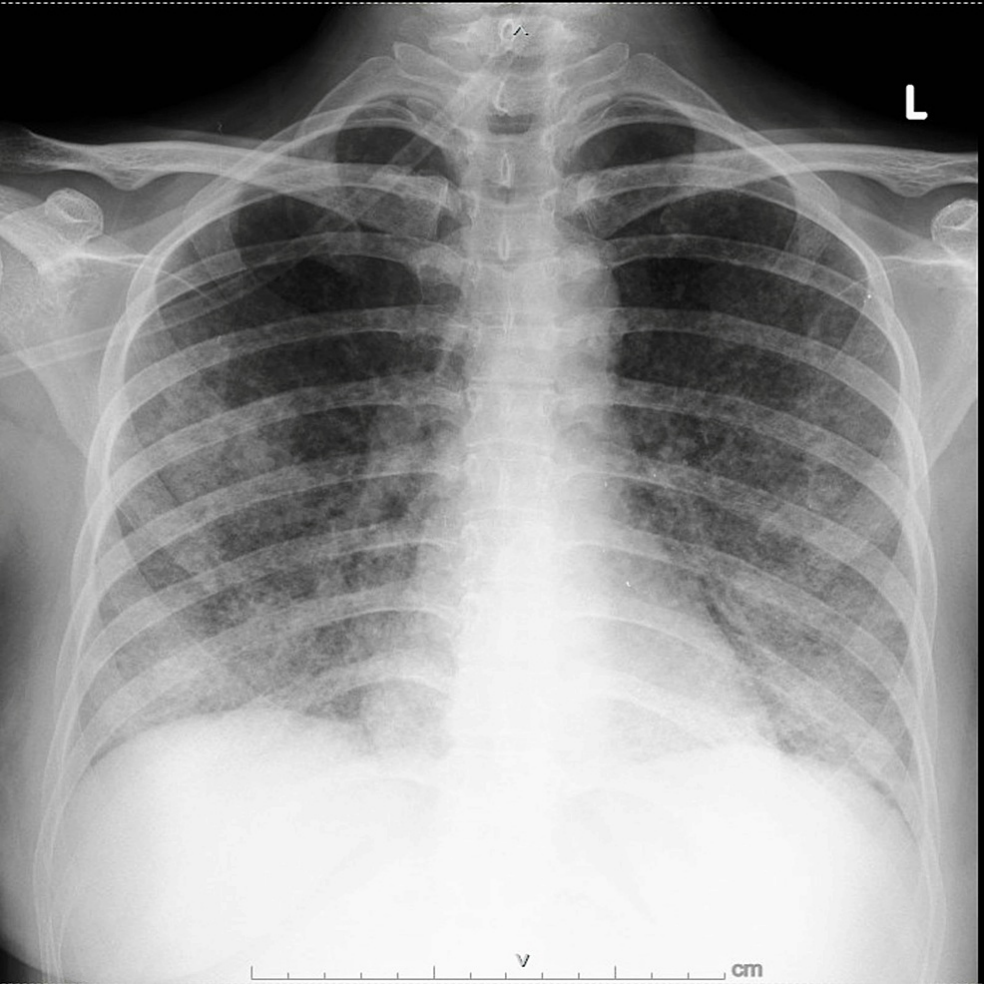
Chest X-ray in the referral hospital

In the referral hospital, a follow-up chest X-ray showed worsening of bilateral opacities (Figure 2). Meropenem, heparin, and FDC anti-tuberculosis drugs were still given with the addition of streptomycin 750 mg OD (im), and a corticosteroid was initiated with methylprednisolone 120 mg OD (iv). The patient was still oxygen dependent with 5 lpm of nasal cannula, but after six days of hospitalization, her condition improved. The patient was discharged in good condition.

## Discussion

Our patient presents with the classical symptoms of COVID-19, which are fever, cough, and shortness of breath. Her clinical symptoms, including a chest X-ray showing bilateral opacities in the peripheral zone, made COVID-19 a top differential diagnosis, especially during the COVID-19 pandemic. The patient was treated for suspected COVID-19 with moderate symptoms. After five days of hospitalization, her symptoms did not improve, and two consecutive RT-PCR swab tests came back negative. We started to think of other differential diagnoses such as infectious causes from bacteria, other viruses (influenza, cytomegalovirus), Pneumocystis jirovecii, fungal, and non-infectious causes such as heart failure, pulmonary embolism, and chronic obstructive pulmonary disease. Tuberculosis as one of the “greatest imitators” in tropical countries also needs to be considered [4,6,7]. However, due to the limited resources and financial constraints, we could not do an extensive work-up and did the test based on our main suspicion. We escalated the empiric antibiotic to meropenem, which has broad-spectrum bacterial coverage, including resistance organisms. We also did an HIV test to rule out immunocompromised states, which can later change our differential diagnosis. Severe infections caused by some organisms, such as cytomegalovirus, Pneumocystis jiroveci, and fungal infections, usually happen in advanced immunocompromised states [8].

It is always a big question: how many negative RT-PCR swab tests are enough to rule out COVID-19? The Indonesian Society of Respirology (ISR) recommends doing two consecutive RT-PCR swab tests in person with clinical signs and symptoms of COVID-19. If the first test was negative, the second test requires two different samples: one sample from the oropharyngeal/nasopharyngeal and one sample from the sputum/bronchial secretion/bronchoalveolar lavage. An alternative diagnosis should be sought if the second RT-PCR swab test is also negative [4]. In this case, we did the third PCR swab test because of our previous experience with a patient with a classical COVID-19 clinical picture and contact history who showed a positive result on the third RT-PCR swab test after two previous negative tests [9]. Although it has a high specificity of 98%, the sensitivity of the RT-PCR swab test is only 60-78%. The optimal number of RT-PCR swab tests conducted before ruling out COVID-19 depends on the COVID-19 prevalence in the area. In the area with a prevalence of 10%, a third negative RT-PCR swab test will give a false negative of 0.75%, while in the area with a prevalence of 25%, a fourth negative RT-PCR swab test will give a false negative of 0.92%, necessitating finding other diagnoses than COVID-19 [10].

Tuberculosis is an infectious disease caused by MTB, presenting mainly as a pulmonary infection. Tuberculosis can be found around the globe, with the main concentration in developing countries [11]. WHO estimated Indonesia as the second most prevalent tuberculosis country, with an incidence rate of 354 per 100,000 people [12]. Patients who get infected with the bacilli for the first time will have primary tuberculosis known as Ghon focus, located mainly in the lower part of the superior lobe or upper part of the inferior lobe of the lung. The Ghon focus will enter a latent state or become a primary, progressive disease. The latent state can be reactivated in the future due to the immunocompromised state forming secondary tuberculosis, mainly located in the apices of the lung. Clinical symptoms of tuberculosis consist of a purulent cough, chronic cough, hemoptysis, and dyspnea. Other constitutional symptoms such as malaise, weight loss, night sweats, and fever can also be noted [11,13]. In our case, after the third negative RT-PCR swab test, we started to think of tuberculosis due to its high prevalence in Indonesia. Rapid molecular testing of the patient's sputum detected MTB. We initiated a four-drug regimen (Isoniazid, Rifampicin, Ethambutol, and Pyrazinamide) in a fixed-drug combination pill, but the symptoms still did not improve. Streptomycin was added to the regimen, and a steroid was given with methylprednisolone 120 mg IV. The rationale for giving a steroid to this patient is that we have already initiated the antituberculosis medication, but her respiratory symptoms were still worsening, so we thought the addition of a steroid could reduce the inflammation alongside her antituberculosis medication. Even though the use of steroids in meningitis and pericarditis has firm evidence, the practice of giving steroids to other types of tuberculosis is still controversial. The use of steroids in severe tuberculosis with respiratory failure has been shown to have benefits regarding mortality reduction. The use of steroids can suppress the cytokine and reduce the aberrant inflammation occurring in patients [14,15]. After six days of treatment in the referral hospital, her condition improved, and she was discharged uneventfully.

## Conclusions

During a pandemic, other diagnoses than COVID-19 should be suspected if the patient had a repeated negative COVID-19 RT-PCR swab. Tuberculosis is one of the main differential diagnoses of patients presenting with respiratory illness in Indonesia and other countries with a high tuberculosis burden.
